# Antimicrobial stewardship in rural and remote primary health care: a narrative review

**DOI:** 10.1186/s13756-021-00964-1

**Published:** 2021-07-13

**Authors:** Jun Wern Yau, Sze Mun Thor, Danny Tsai, Tobias Speare, Chris Rissel

**Affiliations:** 1 Jeffrey Cheah School of Medicine and Health Sciences, Monash University Malaysia, 47500 Bandar Sunway, Malaysia; 2 Flinders University- Rural and Remote Health NT, Royal Darwin Hospital Campus, Rocklands Drive, Tiwi, NT 0810 Australia; 3 Alice Springs Hospital, Central Australian Health Service, Alice Springs, NT 0870 Australia; 4 University of Queensland Centre for Clinical Research, The University of Queensland, Brisbane, QLD Australia

**Keywords:** Antimicrobial stewardship, Inappropriate prescribing, Antimicrobial resistance, Antimicrobial, Bacterial, Primary health care, Anti-infective agents, Rural health, Health education, Public health surveillance

## Abstract

**Background:**

Antimicrobial resistance is an emerging problem worldwide and poses a significant threat to human health. Antimicrobial stewardship programmes are being implemented in health systems globally, primarily in hospitals, to address the growing threat of antimicrobial resistance. Despite the significance of primary health care services in providing health care to communities, antimicrobial stewardship programmes are not well established in this sector, especially in rural and remote settings. This narrative review aims to identify in rural and remote primary health care settings the (1) correlation of antimicrobial resistance with antibiotic prescribing and volume of antibiotic use, (2) appropriateness of antimicrobial prescribing, (3) risk factors associated with inappropriate use/prescribing of antibiotics, and (4) effective antimicrobial stewardship strategies.

**Methods:**

The international literature was searched for English only articles between 2000 and 2020 using specified keywords. Seven electronic databases were searched: Scopus, Cochrane, Embase, CINAHL, PubMed, Ovid Medline and Ovid Emcare. Publication screening and analysis were conducted using Joanna Briggs Institute systematic review tools.

**Results:**

Fifty-one eligible articles were identified. Inappropriate and excessive antimicrobial prescribing and use directly led to increases in antimicrobial resistance. Increasing rurality of practice is associated with disproportionally higher rates of inappropriate prescribing compared to those in metropolitan areas. Physician knowledge, attitude and behaviour play important roles in mediating antimicrobial prescribing, with strong intrinsic and extrinsic influences including patient factors. Antimicrobial stewardship strategies in rural and remote primary health care settings focus on health care provider and patient education, clinician support systems, utility of antimicrobial resistance surveillance, and policy changes. Results of these interventions were generally positive with decreased antimicrobial resistance rates and improved appropriateness of antimicrobial prescribing.

**Conclusions:**

Inappropriate prescribing and excessive use of antimicrobials are an important contributor to the increasing resistance towards antimicrobial agents particularly in rural and remote primary health care. Antimicrobial stewardship programmes in the form of education, clinical support, surveillance, and policies have been mostly successful in reducing prescribing rates and inappropriate prescriptions. The narrative review highlighted the need for longer interventions to assess changes in antimicrobial resistance rates. The review also identified a lack of differentiation between rural and remote contexts and Indigenous health was inadequately addressed. Future research should have a greater focus on effective interventional components and patient perspectives.

**Supplementary Information:**

The online version contains supplementary material available at 10.1186/s13756-021-00964-1.

## Background

Antimicrobials are pharmaceutical agents used to destroy or halt the growth of pathogenic microorganisms and are an integral part of health care. The effectiveness of antimicrobials against a variety of pathogens has been decreasing at alarming rates due to developing antimicrobial resistance (AMR) [1]. Antimicrobial resistance is a global health concern that contributes to patient morbidity and mortality and increases the cost of health care [2]. It has been recognised as one of the most significant health challenges of the present and future, generating global and national responses [3–6].

Antimicrobial stewardship (AMS) programmes combat the rise of AMR through evidence-based multicomponent strategies, including AMR surveillance, education, and guidance to encourage judicious antimicrobial use, to improve health outcomes [7, 8]. AMS programmes are well established in hospital settings, being introduced more than 30 years ago [9]. The implementation of AMS programmes in primary health care (PHC) settings is less well established due in part to additional challenges, including shortages of healthcare professionals and less access to therapeutic tools [10, 11]. Most of the research into effectiveness and implementation of AMS strategies is based in hospital settings. There is a gap in the understanding of how AMS programmes best work in PHC settings, particularly in the rural and remote context.

Around the world, the health of people in rural and remote areas tends to be worse than people in urban centres [12]. ‘Rural’ and ‘remote’ are often use synonymously, however it is important to recognise that these terms describe distinct contexts with differing models of health care [13]. The rurality or remoteness of a location is determined on the basis of geographic remoteness to population centres and relative access to services, which incorporates size of population centres, road distances, integrity of transport infrastructure, and the types and availability of goods and services [14, 15]. While there are different scales to measure remoteness around the world, in the Australian health care context the Modified Monash Model (MMM) considers MMM 4 and 5 as rural and MMM 6 and 7 considered remote and very remote. For example, MMM 4 includes a location in, or within 10 km road distance, of a town with a population between 5000 and 15,000 [14]. The criteria used to define ‘remote’ is a combination of smaller population size, greater road distances to services and other factors, with an exact classification a technical combination of variables [14]. Populations in remote areas tend to be sicker and more dispersed, there are greater workforce shortages, the delivery of health care more costly, and a greater proportion of the population is Indigenous [16]. As the distance from major cities increases, death rates increase, access to health services decline and rates of preventable hospitalisations for chronic diseases markedly rise [17–19]. Recent publications have identified the significant burden of infections and antimicrobial use in remote Australian committees and an upward trend of AMR [11, 20, 21].

This narrative review aims to identify the evidence of the emergence of AMR associated with antimicrobial use and the effectiveness of AMS interventions in the rural and remote PHC setting. There are four objectives of this review: to describe the correlation of AMR with antibiotic prescribing and/or volume of antibiotic use; describe the appropriateness of antimicrobial prescribing in rural and remote PHC; identify risk factors associated with inappropriate use/prescribing of antibiotics; and describe effective AMS strategies in rural and remote PHC settings.

## Methods

Searches were conducted in seven online databases: Scopus, Cochrane, Embase, CINAHL, PubMed, Ovid Medline and Ovid Emcare. Keywords and Boolean operators employed were (‘antimicrobial prescribing’ OR ‘antimicrobial resistance’ OR ‘antimicrobial stewardship’) AND (‘primary health care’ OR ‘primary healthcare’ OR ‘primary care’ OR ‘indigenous’ OR ‘aboriginal’) AND (‘rural’ OR ‘remote’). Only English language articles published between 2000 and 2020 were included to capture studies with greater relevance to current clinical practice and are suitable for thorough analysis.

**Fig. 1:**
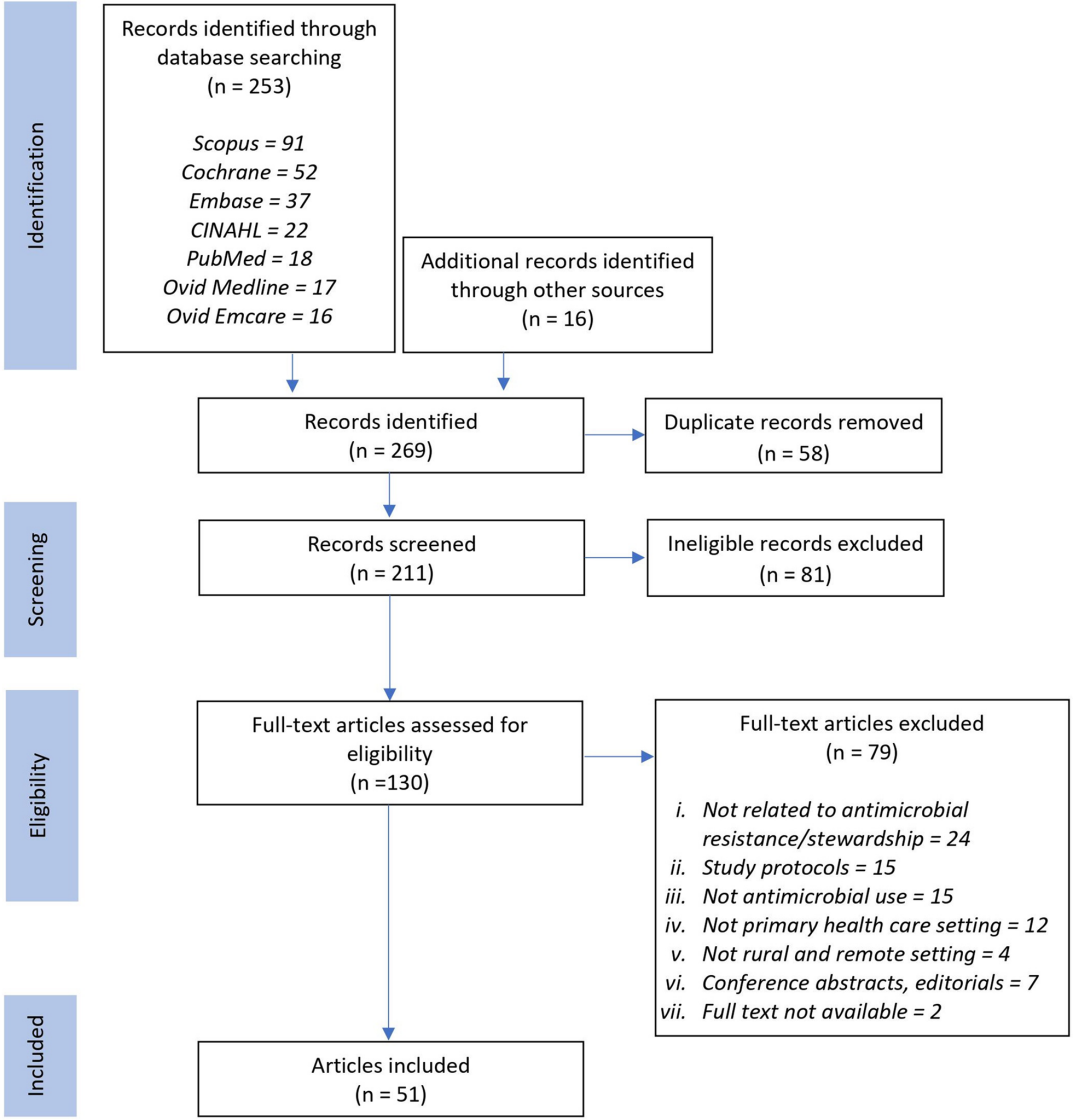
Adapted PRISMA flow chart summarising the search and selection of studies

Records retrieved were independently examined by two authors (JWY and SMT). After the initial selection process, the two authors jointly screened all selected articles and discussed the eligibility of inclusion in this review. Studies which did not feature a remote or rural target group or deemed extraneous were excluded by title and abstract, followed by a full-text review (Fig. 1). Further manual searching for additional relevant articles were performed by checking the reference lists of included studies as well as key scientific texts and source materials.

Data extracted from the included studies were study design, country of research, population under investigation and findings. The main findings were summarised. Additionally, descriptions of interventions and their measured outcomes were included for studies investigating AMS strategies. Subsequently, studies were grouped into four main themes to address the objectives of the review. Key extraction data of each study can be found in Tables 1, 2, 3 and 4. Several studies found to be relevant to more than one theme were categorised in two or more tables. Critical appraisal was performed for all studies using checklists by the Joanna Briggs Institute (JBI), with results classified in Additional file 1: Tables S1–S8. Ethics approval was not sought given that all material used was publicly available and previously published data.

**Table 1:** Correlation of antimicrobial resistance with antibiotic prescribing and/or volume of antibiotic use

Reference #	First author	Year	Study type	JBI checklist used (score/total)	Country	Setting/population	Main findings
*Rural and remote*							
[25]	Doan	2020	Randomized controlled trial	Randomized controlled trial (11/13)	Niger	Children below 5 years of age from 30 villages	Genetic determinants of macrolide resistance were 7.4 times higher at 36 months and 7.5 times higher at 48 months in the azithromycin group compared to placebo. Mass azithromycin distribution also increased determinants of resistance to non-macrolide antibiotics, including 2.1 times higher beta-lactam resistance
[29]	Hare	2013	Prospective cohort study	Cohort Study (10/11)	Australia and Alaska	Indigenous children in outpatient clinics or in hospitals	Azithromycin use was correlated in a ‘cumulative dose–response’ relationship with significantly increased carriage of *S. pneumoniae* and *S. aureus* strains resistant to macrolides. Carriage of *S. aureus* increased but carriage of *S. pneumoniae*, *Haemophilus influenzae* and *Moraxella catarrhalis* decreased with azithromycin use
[23]	Jeong	2020	Retrospective database analysis	Prevalence Study (9/9)	Canada	12 First Nations communities recruited in nursing stations	Skin and soft tissue infections due to community acquired MRSA were highly prevalent in remote, isolated Indigenous communities across Canada, as was use of antibiotics
*Urban*							
[31]	Hoberman	2016	Randomized controlled trial	Randomized Controlled Trial (11/13)	United States	520 children with acute otitis media	Antimicrobial treatment of shorter duration resulted in less favourable health outcomes compared to standard treatment, with no difference in rates of adverse events and antimicrobial resistance
*Mixed urban and rural*							
[32]	Evans	2019	Systematic review	Systematic Review (7/11)	Multiple, including rural Australia	Predominantly children and young people with active trachoma	Communities treated with azithromycin had an approximately fivefold increased risk of resistance at 12 months of *S. pneumoniae*, *S. aureus*, and *Escherichia coli* to azithromycin, tetracycline, and clindamycin, but not to penicillin or trimethoprim-sulfamethoxazole
[24]	Hansen	2019	Systematic review	Systematic Review (10/11)	Multiple, including rural Australia	Varied, both hospitals and primary care	Identification of bacteria resistant to macrolides were more frequent immediately after exposure, but resistance was inconsistent thereafter
[30]	Hare	2015	Randomized controlled trial	Randomized Controlled Trial (11/13)	Australia and New Zealand	Indigenous Australian children living in remote regions and urban New Zealand Māori and Pacific Islander children	At 6 months post-intervention, macrolide resistance declined for *S. pneumoniae* but remained for *S. aureus* strains. Independent factors for macrolide resistance included azithromycin treatment, remote settings and poor adherence

*CI* confidence interval, *MRSA* methicillin-resistant *Staphylococcus aureus*, *OR* odds ratio

**Table 2:** Appropriateness of antimicrobial prescribing in rural/remote primary health

Reference #	First author	Year	Study type	JBI checklist used (score/total)	Country	Setting/population	Main findings
*Rural and remote*							
[59]	Chai	2019	Cross-sectional quantitative study	Cross Sectional Study (8/8)	China	Residents of 12 rural villages	In China, excessive antimicrobial use is prevalent in primary care settings. Use of antimicrobials bought from medicine shops without prescriptions ranged from 8.8 to 17.2% whereas use of antimicrobials leftover from previous illnesses or given by a relative ranged from 7.6 to 13.4%
[34]	Giles	2019	Single-centre, retrospective study	Prevalence Study (5/9)	United States	Rural outpatient family medicine clinic	75% of 28 patients received a first-line antimicrobial based on treatment guidelines, of which 18 obtained a recommended dose. However, an appropriate treatment duration was prescribed for only 17% of patients
[42]	Kwiatkowska	2020	Cross-sectional quantitative study	Cross Sectional Study (7/8)	China	Township health care centre and village clinic	The rural Anhui province in China had considerably high rates of outpatient antibiotic prescribing. While e-records could be useful to inform antimicrobial stewardship, they may have inaccuracies and/or biases
[36]	Sarwar	2018	Cross-sectional quantitative study	Cross Sectional Study (7/8)	Pakistan	16 rural health care centres and 16 basic health units	Antimicrobial agents were frequently prescribed in primary health care centres in Pakistan, a large proportion of which were inappropriate
[38]	Xue	2019	Quasi-experimental	Quasi-experimental Study (9/9)	China	Rural village clinics and township health centres	Primary care providers in rural China frequently prescribed antibiotics inappropriately, predominantly due to deficits in diagnostic knowledge but also to financial incentives linked to drug sales and perceived patient demand
*Mixed urban and rural*							
[44]	Davey	2020	Cross-sectional quantitative analysis	Cross Sectional Study (7/8)	Australia	General practitioners (16% rural)	Recommendations by Australian therapeutic guidelines were adhered when choosing antibiotics. Antibiotic treatment was more likely given to adults than children
[40]	Kumar	2008	Cross-sectional quantitative study	Cross Sectional Study (8/8)	India	Primary and secondary health care settings in rural and urban areas	Higher antibiotic use was correlated with rural settings, lower patient age and higher socioeconomic status. Lower antibiotic prescribing was correlated with government health facilities which have larger allied health support and better infrastructure and specialist practices with more qualified staff
[41]	Kumari Indira	2008	Cross-sectional quantitative study	Cross Sectional Study (7/8)	India	Primary and secondary health care settings in rural and urban areas	Antimicrobials were more commonly prescribed by physicians practising in rural and public/government settings, and to patients presenting with fever and high-income patients
[35]	Rhee	2019	Cross-sectional quantitative study	Cross Sectional Study (7/8)	Kenya	Primary health care facilities in a rural and urban slum	Antibiotics were commonly prescribed inappropriately in the management of diarrhoea in children. Over-prescription was associated with a diagnosis of gastroenteritis in a rural setting and concurrent signs of respiratory infection in an urban setting
[49]	Salm	2018	Questionnaire-based survey	Qualitative Research (8/10)	Germany	Urban and rural general practitioners	While knowledge on bacterial antimicrobial resistance was acceptable, delayed prescription was more commonly adopted by general practitioners in urban areas than those in rural areas
[46]	Silverman	2017	Retrospective database analysis	Prevalence Study (9/9)	Canada	Older patients who presented to a primary care physician with a nonbacterial acute upper respiratory tract infection (8% rural)	Antibiotics were more commonly prescribed by mid- or late-career physicians with high patient volumes as well as those trained outside of the United States or Canada
[39]	Singer	2018	Retrospective cohort study	Cohort Study (8/11)	Canada	32 urban and rural primary care clinics	A potentially inappropriate antimicrobial prescription was given in 18% of primary care visits. For viral infections, older patients, patients with more comorbidities, more office visits and larger or rural practices were more likely to be prescribed antimicrobials inappropriately. For bacterial infections, female patients, younger age and less office visits were more likely
[43]	Staub	2019	Retrospective database analysis	Prevalence Study (7/9)	United States	Retail pharmacies filling outpatient antibiotic prescriptions	Those born in the 1960s and working in rural practices were more likely to be high prescribers, who tend to prescribe broader-spectrum antibiotics
[37]	Wang	2014	Cross-sectional quantitative study	Cross Sectional Study (6/8)	China	39 primary health care facilities (23 city and 16 rural primary health care centres)	Antibiotics were frequently prescribed in primary health care centres in China, a large proportion of which were inappropriate
[56]	Wood	2007	Cross-sectional qualitative study	Cross Sectional Study (6/8)	Wales	General practitioners in practices from urban, post-industrial and rural settings	General practitioners' decision to prescribe a broad-spectrum antibiotic over one with narrow spectrum was influenced by clinical considerations, perceptions of patient expectations and organisational pressures. While both stated their desire to best serve their patients and society, high prescribers were more likely to prioritise immediate needs of patients while average prescribers were more likely to acknowledge long-term consequences
[48]	Yuguero	2019	Cross-sectional qualitative study	Cross Sectional Study (7/8)	Spain	108 general practitioners from 22 primary care centres (54.65% rural)	Prescribing performance was superior in general practitioners who are more empathic, less burned-out, and older

**Table 3:** Risk factors associated with spread of antimicrobial resistance

Reference #	First author	Year	Study type	JBI checklist used (score/total)	Country	Setting/population	Main findings
*Rural and remote*							
[59]	Chai	2019	Cross-sectional quantitative study	Cross Sectional Study (8/8)	China	Residents of 12 rural villages	Use of antimicrobials bought from medicine shops without prescriptions ranged from 8.8 to 17.2% whereas use of antimicrobials leftover from previous illnesses or given by a relative ranged from 7.6 to 13.4%. Antimicrobial prescriptions were less likely to be given to respondents having greater antimicrobial-related knowledge
[45]	Cuningham	2020	Retrospective data analysis	Prevalence Study (9/9)	Northern Australia	15 remote primary health care clinics	The adapted GP NAPS tool demonstrated potential as an audit tool of antimicrobial use for the remote primary health care setting in Australia. Compared with other Australian settings, narrow spectrum antimicrobials were more commonly prescribed with high appropriateness of use (WA: 91%; NT: 82%; QLD: 65%). The dominant treatment indications were skin and soft tissue infections (WA: 35%; NT: 29%; QLD: 40%)
[54]	Chen	2020	Cross-sectional qualitative study	Cross Sectional Study (5/8)	China	Village doctors and township level physicians	The dissonance between physicians' knowledge and their prescribing behaviour were due to various official regulations, institutional pressures to generate revenues, their desire to maintain good patient relationships and concerns for patient safety. Physicians often leave the responsibility for antimicrobial stewardship to the government or higher bodies in the health care system
[38]	Xue	2019	Quasi-experimental	Quasi-experimental Study (9/9)	China	Rural village clinics and township health centres	Primary care providers in rural China frequently prescribed antibiotics inappropriately, predominantly due to deficits in diagnostic knowledge but also to financial incentives linked to drug sales and perceived patient demand
[52]	Zhang	2016	Cross-sectional qualitative study	Cross Sectional Study (7/8)	China	Village doctors, primary caregivers, directors from the local county-level CDC, Health Bureaus or CFDA offices, and township hospital staff	Unnecessary prescribing for children with upper respiratory tract infections was common in village clinics in rural China, where doctors often had inadequate knowledge and misconceptions of antibiotic use. Prescribing behaviour was influenced by doctors' fear of complications, primary caregivers' pressure for antibiotic treatment, and financial considerations of patient retention
*Urban*							
[60]	Collins	2020	Qualitative survey	Prevalence Study (6/10)	United States	Community	Self-prescription of antibiotics should be taken into account in a community-based stewardship programme, in which prescriber education and patient communication should be prioritised. The highest risk of self-prescription was among military personnel, students, immigrants, isolated and rural populations, and uninsured patients
*Mixed urban and rural*							
[50]	Al-Homaidan	2018	Cross-sectional qualitative study	Cross Sectional Study (6/8)	Saudi Arabia	20 rural and 12 urban primary health cares	Many physicians believed that antibiotic use lessens symptoms in viral disease, and attributed bacterial resistance to inadequate prescription, use without prescription, and patient non-compliance. The pharmacist was often blamed for contributing to antibiotic resistance. High fever was regarded as the symptom prompting antibiotic prescription when laboratory confirmation was unavailable
[58]	Barker	2017	Cross-sectional qualitative study	Cross Sectional Study (6/8)	India	Community members in 3 rural and 2 urban villages	Community members' understanding of antibiotics and consequences of misuse were low
[51]	Dallas	2014	Qualitative survey	Prevalence Study (8/10)	Australia	Rural and urban general practice registrars	General practice registrars recognised that evidence-based antibiotic prescribing is important and overprescribing leads to potentially increased resistance. However, discrepancy between their knowledge and behaviours exist because of patient and system factors, diagnostic uncertainty, transitioning from hospital medicine, and the habits of, and relationship with, their supervisor. Some registrars opined that some specific antibiotic would not contribute to resistance patterns
[53]	Duane	2015	Qualitative study	Qualitative Research (8/10)	Ireland	General practice and community setting	Formal feedback on prescribing was seldom given to general practitioners and most were unfamiliar with local resistance patterns. Instead, antibiotic prescribing practices were formed through habit, anecdotal evidence from patient observation, and the individual laboratory results
[57]	Fletcher-Lartey	2016	Cross-sectional qualitative study	Cross Sectional Study (5/8)	Australia	Primary care general practitioners (37.5% rural)	General practitioners cited patient expectations, which includes limited time, poor doctor–patient communication and diagnostic uncertainty, as the primary reason for prescribing inappropriately. Many did not attribute their prescribing in primary care to the development of antibiotic resistance, unlike use in hospitals or for veterinary purposes
[40]	Kumar	2008	Cross-sectional quantitative study	Cross Sectional Study (8/8)	India	Primary and secondary health care settings in rural and urban areas	Higher antibiotic use was correlated with rural settings, lower patient age and higher socioeconomic status. Lower antibiotic prescribing was correlated with government health facilities which have larger allied health support and better infrastructure and specialist practices with more qualified staff
[40]	Kumari Indira	2008	Cross-sectional quantitative study	Cross Sectional Study (7/8)	India	Primary and secondary health care settings in rural and urban areas	Antimicrobials were more commonly prescribed by physicians practising in rural and public/government settings, and to patients presenting with fever and high-income patients
[55]	Nair	2019	Cross-sectional qualitative study	Cross Sectional Study (4/8)	India	Allopathic doctors, informal health providers, nurses, and pharmacy shopkeepers	Doctors did not translate knowledge into practice as many prescribed antibiotics inappropriately, citing inconsistent follow up, lack of testing facilities, risk of secondary infections, and unhygienic living conditions as their reasons to prescribe. Prescription behaviour was influenced by patients demanding antibiotics and seeking the fastest cure possible. Allopathic doctors and informal health providers frequently impart blame on the other party for contributing to antibiotic resistance, and yet both referred patients to one another
[61]	Salm	2018	Cross-sectional quantitative survey	Cross Sectional Study (8/8)	Germany	Rural, suburban and urban populations	Recent antibiotic use likely confers patients with more knowledge, highlighting health literacy as a tool against inappropriate antibiotic use
[39]	Singer	2018	Retrospective cohort study	Cohort Study (8/11)	Canada	32 urban and rural primary care clinics	A potentially inappropriate antimicrobial prescription was given in 18% of primary care visits. For viral infections, older patients, patients with more comorbidities, more office visits and larger or rural practices were more likely to be prescribed antimicrobials inappropriately. For bacterial infections, female patients, younger age and less office visits were more likely
[47]	Wang	2020	Cross-sectional quantitative study	Cross Sectional Study (8/8)	China	67 primary care facilities (19 urban, 48 rural)	Prescribers' insufficient knowledge, indifference to changes, complacency with satisfied patients, low household income and rural location coincided with higher antibiotic use

*CDC* Centres for Disease Control and Prevention, *CFDA* China Food and Drug Administration, *GP NAPS* General Practice version of the National Antimicrobial Prescribing Survey, *NT* Northern Territory, *QLD* Queensland, *WA* Western Australia

**Table 4:** Antimicrobial stewardship strategies in remote primary health care settings

Reference #	First author	Year	Study type	JBI checklist used (score/total)	Country	Participants (number, sites)	Intervention description	Outcome measures	Main findings
*Patient and provider education*									
*Rural and remote*									
[70]	Belongia	2001	Non-randomised, controlled trial	Quasi-experimental Study (8/9)	United States	Parents and 151 primary care clinicians who provide paediatric care in a rural area	Clinician and community education via educational meetings and printed educational materials	Number of solid and liquid prescriptions per clinician, retail antibiotic sales, nasopharyngeal carriage of penicillin-nonsusceptible *Streptococcus pneumoniae*	Median number of solid antibiotic prescriptions per clinician decreased 19% in the intervention region and 8% in the control region. Median number of liquid antibiotic prescriptions per clinician decreased 11% in the intervention region but increased 12% in the control region. Retail antibiotic sales dropped in the intervention region but not in the control region
[62]	Chiswell	2019	Retrospective pretest–posttest study	Quasi-experimental Study (7/9)	United States	207 ‘walk-in’ patients in a rural primary care practice diagnosed with a respiratory tract infection	One-year patient education intervention programme involving repeated exposure to posters and handouts containing relevant health information	The number of antibiotics prescribed for respiratory tract infections	Antibiotic prescription rate decreased from 56.3% in the preintervention group to 28.8% in the postintervention group (x^2^ = 15.97, *P* < 0.001). The number of immediate antibiotic prescriptions dropped from 31.1% in the preintervention group to 13.5% in the postintervention group (x^2^ = 9.28, *P* < 0.05)
[65]	Wei	2017	Cluster randomised controlled trial	Randomized Controlled Trial (11/13)	China	Children aged 2–14 years given a prescription following a primary diagnosis of an upper respiratory tract infection in 25 primary care township hospitals across 2 rural counties	Clinician guidelines and training on appropriate prescribing, monthly prescribing peer-review meetings, and brief patient/caregiver education	Primary outcome: Antibiotic prescription rate Secondary outcomes: Rates of prescribing multiple antibiotics, broad-spectrum antibiotics and intravenous antibiotics, proportion of prescriptions containing nonantibiotic medicines, cost	Antibiotic prescription rate decreased from 82 to 40% in the intervention group, and from 75 to 70% in the control group, yielding an absolute risk reduction in antibiotic prescribing of − 29% (95% CI − 42 to − 16; *P* = 0.0002)
[66]	Wei *(subgroup follow-up analysis of above study)*	2019	Cluster randomised controlled trial	Randomized Controlled Trial (11/13)	China	Children aged 2–14 years given a prescription following a primary diagnosis of an upper respiratory tract infection in 14 primary care township hospitals across 1 rural county	Clinician guidelines and training on appropriate prescribing, monthly prescribing peer-review meetings, and brief patient/caregiver education	Primary outcome: Antibiotic prescription rate Secondary outcomes: Factors in sustaining intervention, rates of prescribing multiple antibiotics, broad-spectrum antibiotics and intravenous antibiotics, proportion of prescriptions containing nonantibiotic medicines, cost	The APR difference in the intervention arm at 6 months is − 49% (95% CI − 63 to − 35; *P* < 0.0001) compared to baseline. Compared to baseline, the APR difference in the intervention arm at 18 months is − 36% (95% CI − 55 to − 17; *P* < 0.0001). Compared to that at 6 months, the difference at 18 months represented no change in the APR. Factors sustaining reductions included doctors’ improved knowledge and communication skills and focused prescription review meetings, whereas lack of supervision and monitoring may be associated with relapse
[68]	Cummings	2020	Quasi-experimental	Quasi-experimental Study (6/9)	United States	Rural urgent care centres	Three behavioural interventions: (1) physician and patient education via lectures, presentations, media and distributable materials, (2) public commitment from the Medical Director of Urgent Care, and (3) peer comparison via individual feedback and blinded ranking emails	Proportion of acute respiratory tract infection diagnosis visits that received an inappropriate antibiotic	Percentage of inappropriate prescribing decreased 14.9%, from 72.6 to 57.7% (95% CI − 20.30% to − 9.05%; *t*, 5.44; *P* < 0.0001). Interrupted time series analysis showed a significantly lowered rate of antibiotic-inappropriate prescribing (95% CI − 4.59 to − 0.59; *P* = 0.014)
[74]	Zhang	2018	Cluster randomised controlled trial	Randomized Controlled Trial (11/13)	China	Children aged 2–14 years given a prescription following a primary diagnosis of an upper respiratory tract infection in 25 primary care township hospitals across 2 rural counties	Clinician guidelines and training on appropriate prescribing, monthly prescribing peer-review meetings, and brief patient/caregiver education	Cost per percentage point decrease in the antibiotic prescription rate	Incremental cost of US$0.03 per percentage point reduction in antibiotic prescribing
*Mixed urban and rural*									
[67]	Varonen	2007	Randomised controlled trial	Randomized Controlled Trial (8/13)	Finland	30 rural and urban health centres	Nationwide guidelines implementation programme involving education based on a PBL or AD method facilitated by local general practitioners	Compliance with acute maxillary sinusitis management in national Current Care guidelines	Slight increase in the use of the first-line drug amoxicillin (39–48% in AD centres, 33–45% in PBL centres, controls 40%). Proportion of antibiotic courses with recommended duration increased (34–40% in AD centres, 32–47% in PBL centres, controls 43%)
[73]	Little	2001	Randomised controlled trial	Randomized Controlled Trial (9/13)	England	315 children presenting with acute otitis media in 42 general practices (33% mixed urban and rural settings)	Two treatment strategies – immediate antibiotics or delayed antibiotics – supported by standardised advice sheets	Resolution of symptoms, absence from school or nursery, paracetamol consumption	Children prescribed antibiotics immediately had shorter illness [− 1.1 days (95% CI − 0.54 to − 1.48)], fewer nights disturbed (− 0.72 (95% CI − 0.30 to − 1.13)], and slightly less paracetamol consumption [− 0.52 spoons/day (95% CI: − 0.26 to − 0.79)], but had higher incidence of diarrhoea (14/150 (9%) v 25/135 (19%), x^2^ = 5.2, *P* = 0.02), compared to delayed prescription
[75]	Haenssgen	2018	Quasi-experimental qualitative study	Quasi-experimental Study (7/9)	Laos	1130 peri-urban villagers	A one-off educational activity comprising of six sections—a mapping exercise, a medicine matching game, a resistance game, a role-play activity, a healthy-wealthy game and a feedback session	Attitudes and knowledge on antibiotics, treatment-seeking behaviour, and social networks	Awareness and understanding of antibiotic resistance improved, but effects on attitudes were minor. Mixed impact on behavioural changes. Activity-related communication spread within groups of greater privilege
*Physician support systems*									
*Rural and remote*									
[64]	Samore	2005	Cluster randomised trial	Randomized Controlled Trial (7/13)	United States	407,460 inhabitants and 334 primary care clinicians in 12 rural communities	6 communities received a community intervention alone and 6 communities received community intervention plus CDSS targeted toward primary care clinicians	Community-wide and diagnosis-specific antimicrobial usage	Prescribing rate decreased from 84.1 to 75.3 per 100 person-years in the CDSS arm vs 84.3 to 85.2 in community intervention alone (*P* = 0.03). Antimicrobial prescribing for visits in the antibiotics “never-indicated” category during the postintervention period decreased 32% in CDSS communities and 5% in community intervention-alone communities (*P* = 0.03). Macrolide use decreased significantly in CDSS communities (*P* = 0.001) but not in community intervention–alone communities
[69]	Gonzales	2013	Cluster randomised controlled trial	Randomized Controlled Trial (9/13)	United States	33 primary care practices in a rural region	Simple clinical algorithm implemented via a traditional printed decision support (PDS) or a computer-assisted decision support (CDS) strategy integrated into the workflow of an electronic health record	Antibiotic prescription rates for uncomplicated acute bronchitis	Percentage of antibiotic prescription decreased compared to baseline at the PDS intervention sites (from 80.0 to 68.3%) and at the CDS intervention sites (from 74.0 to 60.7%) but increased slightly at the control sites (from 72.5 to 74.3%). Differences due to interventions were statistically significant from the control sites (*P* = 0.003 and *P* = 0.01 for PDS and CDS, respectively) but not between themselves (*P* = 0.67)
[72]	Rubin *(Extension analysis of study described in Reference #64)*	2006	Observational quantitative study	Randomized Controlled Trial (7/13)	United States	99 primary care providers serving rural communities	A standalone personal digital assistant-based CDSS tool for the diagnosis and management of acute respiratory tract infections	Usage patterns and acceptability of the tool	Adherences with CDSS recommendations for the five most common diagnoses and for antibiotic choice were 82% and 76%, respectively. Logistic regression models indicate that provider adherence improved with each ten cases entered into the system (*P* = 0.001). Respondents believed the CDSS was easy to use, and most (44/65; 68%) reported that patient encounters were either the same duration or slightly faster, when using the CDSS tool compared with their usual practice
[76]	Madaras-Kelly	2006	Experimental quantitative cohort study	Quasi-experimental Study (8/9)	United States	192 patients visiting 2 rural community pharmacies for broad-spectrum antibiotics	Community pharmacists conducted guided interviews regarding patient symptoms and intercepted inappropriate prescriptions through communication with the ordering clinician to decrease broad-spectrum antibiotic use in upper respiratory infections	Number of patients agreeable for interview, pharmacist time, primary care provider acceptance of the recommendations, and patient opinion data regarding the pharmacy intervention	3% of the patients who were approached declined to discuss their symptoms and treatment with the pharmacist. 7% (*n* = 4) of patients permitted the community pharmacist to contact the prescriber to discuss first-line therapeutic alternatives. 2 of 3 clinicians contacted by pharmacists were receptive to altering the broad-spectrum antimicrobial to first-line antimicrobial therapy
*Surveillance*									
*Rural and remote*									
[45]	Cuningham	2020	Retrospective data analysis	Prevalence Study (9/9)	Northern Australia	15 remote primary health care clinics	The General Practice version of the National Antimicrobial Prescribing Survey (GP NAPS) tool modified for remote primary health care clinics	Antimicrobials used, indications and the treating health professional to yield similarities and differences in prescribing patterns, appropriateness of antimicrobial use and functionality of the GP NAPS tool	The adapted GP NAPS tool demonstrated potential as an audit tool of antimicrobial use for the remote primary health care setting in Australia. Compared with other Australian settings, narrow spectrum antimicrobials were more commonly prescribed with high appropriateness of use (WA: 91%; NT: 82%; QLD: 65%). The dominant treatment indications were skin and soft tissue infections (WA: 35%; NT: 29%; QLD: 40%)
[77]	Hui	2015	Mathematical model	Diagnostic Test Accuracy Test (7/10)	Australia	Simulated remote indigenous community	Individual-based mathematical model to determine the impact of molecular testing on AMR surveillance of gonorrhoea	Time delay between first importation and the first confirmation that the prevalence of gonorrhoea AMR has breached the 5% threshold (when a change in antibiotic should occur)	In the best-case scenario, the alert would be triggered within 3–6 months of the resistance proportion exceeding the 5% threshold, at least 8 months earlier than using culture alone
*Mixed urban and rural*									
[78]	Schwartz	2019	Database validation	Diagnostic Test Accuracy Test (6/10)	Canada	9272 physicians prescribing antibiotics to patients ≥ 65 years in urban (90.3%) and rural (9.7%) locations of practice	IQVIA Xponent database of dispensed antibiotic prescription counts aggregated at the physician prescriber-level	Agreement and correlation between Xponent and Ontario Drug Benefit database, performance characteristics for Xponent to accurately identify high prescribing physicians	The Xponent database has a specificity of 92.4% (95% CI 92.0–92.8%) and PPV of 77.2% (95% CI 76.0–78.4%) for correctly identifying the top 25th percentile of physicians by antibiotic volume. In the sensitivity analysis, 94% of the top 25th percentile physicians in Xponent were within the top 40th percentile in the reference database. The mean number of antibiotic prescriptions per physician were similar, but the error was greater in rural areas
*National policies*									
*Mixed urban and rural*									
[63]	Hammond	2020	Ecological retrospective database analysis	Prevalence Study (6/9)	United Kingdom	163 urban (80.61%) and rural (14.12%) primary care practices	Incentivising reduced primary care prescribing of co-amoxiclav, cephalosporins and quinolones for any infection	Primary care antibiotic dispensing and antibiotic resistance in community-acquired urinary *Escherichia coli*	Overall antibiotic dispensing per 1000 registered patients decreased 11%. Antibiotic reductions were associated with reduced within quarter antibiotic resistance to amoxicillin, ciprofloxacin and trimethoprim, reduced subsequent quarter resistance to trimethoprim and amoxicillin, and increased within and subsequent quarter resistance to cefalexin and co-amoxiclav
[68]	Yin	2018	Retrospective database analysis	Prevalence Study (6/9)	China	500 secondary and tertiary hospitals, 600 urban PHC centres and 1600 rural PHC centres	Zero mark-up policies and national policy to improve the rational use of antibiotics in primary health care centres	Data on total and specific antibiotic consumption	Overall antibiotic consumption increased from 12.859 DID in 2012 to 15.802 DID in 2014. When national policies were introduced, this decreased to 13.802 DID in 2016. After an upward trend for 3 years, oral and parenteral antibiotic consumption decreased in rural PHC centres by 12% and 33% from 2014 to 2016

*AD* academic detailing, *AMR* antimicrobial resistance, *APR* antibiotic prescription rate, *CDS* computer-assisted decision support, *CDSS* clinical decision-support system, *CI* confidence interval, *DID* DDD per 1000 inhabitants per day, *GP NAPS* General Practice version of the National Antimicrobial Prescribing Survey, *NT* Northern Territory, *PBL* problem-based learning, *PDS* printed decision support, *PHC* primary health care, *PPV* positive predictive value, *QLD* Queensland, *WA* Western Australia

## Results

### Study selection process

The database search identified 253 articles, of which 58 were duplicates. An additional 16 articles were identified through manual searches. The titles and abstracts of 211 articles were screened, with 81 articles excluded due to not meeting the eligibility criteria. Full-text analysis was performed for 130 studies, of which 79 were excluded to yield a final total of 51 articles for inclusion in this review. Reasons of exclusion include irrelevancy to AMS, AMR or antimicrobials in general as well as studies conducted in hospital settings. Figure 1 illustrates the search and selection process.

### Correlation of antimicrobial resistance with antibiotic usage and/or volume of antibiotic use

The link between antimicrobial use and AMR continues from the hospital into the PHC setting, as evidenced by various studies across the globe. An ecologic analysis of data from 20 countries demonstrated that antibiotic use exerted selection pressure that has directly increased the resistance of multiple known pathogenic types of *Streptococcus sp.* [22]. Similarly, a chart review of Canadian Indigenous communities found methicillin-resistant *Staphylococcus aureus* (MRSA) to be the organism responsible for over 40% of skin and soft tissue infections as well as a high prevalence of antibiotic use in these communities, although direct correlations between the two phenomena could not be made [23]. Further evidence can be found in a systematic review by Hansen et al. [24], where immediately following macrolide exposure, resistant bacteria were identified in participants with greater frequency. Although later trends were subsequently met with inconsistency, resistance is regarded as a major adverse effect of macrolide treatment [24]. This is substantiated by a study by Doan et al. [25] involving children below 5 years, in which mass azithromycin administration resulted in up to 7.5 times (95% confidence interval [CI] 3.8–23.1) higher genetic determinants of resistance to macrolides at 48 months. Costelloe et al. [26] demonstrated that antibiotic resistance is highest in the month following treatment, with effects lasting up to a year. This systematic review presented pooled odds ratio (OR) for resistance among urinary tract bacteria of 2.5 (95% CI 2.1–2.9) and 1.33 (95% CI 1.2–1.5), and among respiratory tract bacteria of 2.4 (95% CI 1.4–3.9) and 2.4 (95% CI 1.3–4.5) within 2 and 12 months of antibiotic treatment, respectively [26].

Prescribing practices in PHC play a major influencing role in AMR rates. Prescription, dispensation and use of penicillin, especially classes susceptible to beta-lactamase, were associated with increased risks of nasal carriage of resistant *S. aureus,* with ORs of 1.18 (95% CI 1.04–1.35) and 1.09 (95% CI 1.00–1.18) according to two large cross-sectional studies [27, 28]. Azithromycin use was likewise correlated with nasal carriage of *S. pneumoniae* and *S. aureus* strains which are resistant to macrolides [29, 30]. Hare et al. also discovered a dose–response relationship between azithromycin use and carriage of macrolide-resistant *S. pneumoniae* and *S. aureus* strains, which were found in 21% (95% CI 14–28) and 1% (95% CI 0–5) respectively when azithromycin was not used, 28% (95% CI 17–38) and 14% (95% CI 6–22) when azithromycin was used infrequently, and 38% (95% CI 26–49) and 23% (95% CI 14–33) when azithromycin was used frequently (nptrend *P* = 0.002 and *P* < 0.001 respectively) [29]. The systematic review by Costelloe et al. [26] accentuated this relationship by drawing associations between multiple or longer durations of antibiotic courses with higher AMR rates, especially for amoxicillin and trimethoprim. However, a randomised controlled trial (RCT) that included 520 children with acute otitis media found that a shorter amoxicillin–clavulanic acid antibiotic regimen of 5 days did not affect AMR rates (*P* = 0.58) and instead, produced less favourable health outcomes with greater likelihood of clinical failure and worse mean symptom scores (*P* = 0.001) compared to the standard 10-day regimen [31]. Nevertheless, this study has limited generalisability due to the age group and disease type [31].

Risks for AMR development are not limited to the specific antimicrobial agent used to treat a particular infection, as suggested in a recent revision of a Cochrane systematic review on the mass treatment of trachoma using antibiotics [32]. Here, evidence shows with high certainty that *S. pneumoniae*, *S. aureus* and *Escherichia coli* exhibited approximately fivefold increase in resistance at 12 months not only to agents used to treat trachoma, namely azithromycin and tetracycline, but also to clindamycin despite having no role in trachoma management [32]. Likewise, Doan et al. [25] found that beta-lactam resistance determinants were 2.1 times (95% CI 1.2–4.0) higher after mass azithromycin distribution. Cross-resistance is a genuine cause for concern, as it expedites the development of multidrug-resistant pathogens, thereby rendering progressively more lines of treatment ineffective.

Overall, the emergence of AMR in the PHC setting has been associated with high-volume antibiotic prescribing and use. Therefore, sustained, large-scale efforts are required to regulate such practices.

### Appropriateness of antimicrobial prescribing in rural and remote primary health care

Inappropriate prescribing is generally defined as the prescription of antimicrobial agents that do not adhere to authorised guidelines in terms of type of antimicrobial chosen, dose and/or duration, or are deemed unnecessary [33]. In rural and remote areas, inappropriate prescribing was reported to be highly prevalent, with 18–88% of antimicrobial prescriptions deemed inappropriate depending on the country [34–38]. Antibiotics were the antimicrobial agents most frequently incorrectly prescribed [35–39].

Clinicians in rural and remote PHC services tended to be high-volume prescribers of antimicrobial agents [40–43]. Antibiotics were prescribed more frequently in rural regions compared to urban areas, 85% versus 68–80% respectively [40–42]. Primary health care services in rural and remote areas face additional challenges in the provision of health care, including limited resource allocations and a different patient population compared to urban clinics, which may contribute to differences in prescribing.

An Australian study showed no significant difference in prescribing behaviour and adherence to guidelines between rural- and urban-based physicians [44]. While this study portrayed a positive outlook in Australia, the results cannot be generalised as the focus was on early-career general practitioners and urinary tract infections only, and rural practices constituted only 16% of the study locations [40]. An audit of antimicrobial use in 15 remote PHC clinics across three states in northern Australia found that appropriateness was high compared to general practices in urban settings [45]. Approximately 91%, 82% and 65% of antimicrobial use in Western Australia, Northern Territory and Queensland adhered to clinical guidelines, endorsed by experts, or constituted agents with the narrowest targets. This appreciably high level of appropriateness was demonstrated by 86% of nurses and 73% of doctors in this region [45]. The high rates of appropriateness in this study may be a result of government endorsed clinical guidelines that the nurses are legally bound to comply with in order to prescribe.

Measures of prescribing appropriateness are highly dependent on specific diseases and clinical guidelines used. For example, a study on childhood diarrhoea by Rhee et al. [35] designated the presence of dysentery as the standard for antibiotic prescription, with over-prescription defined as antibiotics given for non-dysentery cases and under-prescription as absence of antibiotics for children with dysentery. In some instances, prescribing appropriateness were inferred directly from antimicrobial use, particularly antibiotics prescriptions for infections not commonly suspected to be of bacterial origin such as acute upper respiratory tract infections [46]. Often, an antimicrobial prescription is deemed appropriate if it is in accordance with local or international therapeutic guidelines designed for that disease entity [35, 44]. Nevertheless, an Australian study assessed both prescribing appropriateness and guideline adherence, which accounted for prescriptions deviating from guidelines for a justified clinical reason [45].

### Risk factors associated with inappropriate use/prescribing of antibiotics

Rurality is often found to be an independent risk factor for inappropriate prescribing [30, 39–41, 47]. Rural and remote areas have widely distributed populations who generally experience poorer health and who are serviced by fewer doctors per population [39]. This invariably translates to increased physician fatigue due to high workloads, which is associated with excessive and error-prone prescribing [46, 48]. Despite having AMR awareness, rural clinicians were less likely to adopt steps to reduce antibiotic prescriptions, as a survey by Salm et al. [49] suggested.

Aside from the remoteness of practice mentioned above, several other factors contribute to the overuse and misuse of antimicrobials, which ultimately play a major role in the emergence of AMR. Chiefly, physician knowledge and behaviour exert a strong influence on antimicrobial use in the population. Numerous studies have found insufficient awareness of the importance of judicious prescribing and lack of knowledge on local resistance patterns to be major drivers of AMR [38, 47, 50–53]. Most frequently, physicians prescribe antibiotics for viral infections, indicating a substantial need for clinician education [39, 50]. This problem is not unique among clinicians in rural and remote settings, as those in urban primary care practices share similar knowledge, attitudes and practices [50, 51, 53].

Although many rural-based physicians commonly recognised the negative consequences of over-prescription, some nevertheless continue to prescribe antimicrobials liberally to patients [38, 51, 54]. This can be due to both internal and external reasons. Doctors cited apprehension of medical complications from undertreatment, perceived patient expectations and intentions to preserve good relations, and the notion that selected prescribed antibiotics would not contribute to resistance as grounds for discrepancy in personal knowledge and clinical practice [38, 51, 52, 54–57]. Concurrently, external pressures including financial considerations in line with incentivising drug sales and patient retention may compel physicians into prescribing inappropriately [38, 52, 54]. System factors such as insufficient accessibility to follow-up as well as the fear of litigation or related medico-legal issues were also strong drivers of excessive antibiotic prescribing [51, 54].

Furthermore, attitudes of physicians may influence the development of AMR in rural and remote settings. It is possible that some PHC physicians are complacent, prefer to prescribe by habit, or are reluctant to keep up with new recommendations in prescribing [47, 53]. Other health care professionals such as pharmacists and informal health providers may contribute to AMR [50, 55] particularly in countries and regions where there is relatively unrestricted access to antimicrobials.

Patient factors are also identified as playing a role in the development of AMR. In particular, non-compliance to prescribed antimicrobial treatments, especially poor adherence to the full course of antibiotics, facilitates the development of AMR in the community [50, 58]. Treatment decisions are influenced by patient demands, and often driven by unfounded beliefs of obtaining the ‘fastest cure’ [55]. In some countries and regions where antimicrobial dispensing is not strictly regulated, members of the public may self-prescribe antimicrobial agents for ailments [50, 58–60]. The inappropriate use of antibiotics and demands on clinicians to provide antibiotics may result from a poor understanding of the implications of their misuse, limited access to appropriately trained doctors, scarce resources and limited health literacy [58, 61].

Patient attributes associated with inappropriate prescribing and higher AMR rates include patients of a younger age group, female gender, with other co-morbidities and higher socioeconomic status [28, 39]. While further studies are required to elucidate the mechanisms and processes behind these correlations, it is postulated that they could be explained by greater anxiety levels in these populations. For example, parents desire a rapid relief of symptoms for their children, and patients of a higher socioeconomic strata can have greater demands [40, 41]. Patient expectations in relation to antibiotic treatment was identified by physicians, as placing undue pressure on the prescription of antimicrobials to satisfy patients [57].

### Antimicrobial stewardship strategies in remote and rural primary health care settings and their effectiveness

A total of 16 interventions are included in this section under four distinct themes according to their intervention focus, with further detail outlined in Table 4. Although the measured outcomes exhibited a high degree of heterogeneity among the studies, a few main groupings can be elucidated from the pooled data, namely rates and appropriateness of antimicrobial prescribing. Nine studies had antimicrobial prescription rates as their primary outcome measure [62–70]. Of these studies, seven had significantly lowered prescription rates post intervention [62–66, 68–70], in which results ranged from an 11% decrease when the United Kingdom incentivised reduced prescribing of specific antibiotics [63] to a 49% drop when a multi-dimensional clinician-focussed intervention was implemented in China [66]. A total of four studies reported on appropriateness of prescribing [64, 67, 71, 72], and all but one—the MIKSTRA study [67]—demonstrated meaningful improvements after intervention.

#### Health care provider and patient education

Three publications focused on the provision of education to health care providers, where clinicians and supporting staff received training on antimicrobial prescribing, including implementation of antimicrobial stewardship guidelines and strategies such as delayed prescriptions [65, 67, 73]. Although immediate antibiotic prescription reduced symptom severity and duration of acute otitis media in children by about a day, these benefits were marginal as symptoms were already improving after 24 hours [73]. This study also found that doctors’ perception of patient expectations were often overestimated—most parents were satisfied with delayed antibiotic prescribing for their child’s illness [73]. A large RCT in rural China implemented a comprehensive clinician-oriented intervention which consisted of clinical guideline implementation/enforcement, monthly reviews on physicians’ prescribing rates, training on communication skills and brief education for caregivers [65]. Besides proving to be highly cost-effective [74], this multifaceted education strategy successfully decreased antibiotic prescribing rates for childhood upper respiratory tract infections by 29% (*P* = 0.0002) at 6 months [65] and up to 36% (*P* < 0.0001) at 18 months by virtue of improved physician knowledge and communication with patients [66].

On the other hand, modest improvements to antibiotic prescribing were observed in the Finnish MIKSTRA study which promoted the use of first-line treatment for the treatment of acute maxillary sinusitis according to national guidelines recommendations [67]. An education programme using two training methods facilitated by local general practitioners were employed, which were: problem-based learning (PBL) based on group work and an academic detailing (AD) process involving various information sources, feedback and external visits [67]. The RCT only managed to yield minor positive effects on antibiotic prescribing with use of the first-line drug amoxicillin and appropriate duration of antibiotic courses, with the former increasing from 33 to 45% and 39 to 48% while the latter increased from 32 to 47% and 34 to 40% in PBL and AD centres, respectively. These shortcomings were likely due to various resource issues throughout the trial, which included physician shortages in rural PHCs and improper adherence to guidelines brought about by education methods which did not adequately address the local context [67].

Patient education was investigated by a quasi-experiment conducted among Lao villagers by Haenssgen et al. [75] in which a one-off education session involving interactive health-based activities was implemented with mixed results, especially on behavioural change towards antibiotic use. There were noticeable improvements in awareness and understanding of antibiotic resistance, but not attitudes. A combined approach involving education of both the physician and the patient using presentations and printed materials on relevant health information was explored by three studies, all of which showed significant reductions in undesirable outcomes [62, 70, 71]. Chiswell et al. and Belongia et al. decreased antibiotic prescription rates in the rural community, with the former even lowering immediate antibiotic prescriptions from 31.1 to 13.5% (*P* < 0.05) [62, 70]. Cummings et al. reduced the proportion of antibiotic-inappropriate prescriptions by 14.9% (95% CI − 20.30% to − 9.05%; *P* < 0.0001) via distribution of education materials as well as clinician peer comparisons of prescribing behaviour [71].

#### Physician support systems

The effectiveness of a clinical-decision support system (CDSS) was researched in two RCTs in rural areas of the United States: Samore et al. [64] and Gonzales et al. [69]. Different implementations of CDSS—based on paper and electronic tools—were employed and compared, and an extension study was performed in the former focussing on a CDSS system based on personal digital assistants [72]. These CDSS-based interventions were effective at mitigating prescriptions for conditions not requiring antibiotics by 32% in one study [60] and between 11.7 and 13.3% in the other [69]. CDSS also resulted in an increase of 2.7% (*P* = 0.016) in provider adherence to guideline recommendations [72].

A cohort study by Madaras-Kelly et al. [76] described an intervention carried out by rural community pharmacists in which patients were interviewed and the prescribing clinicians contacted before dispensing of a broad-spectrum antibiotic. This intervention did not attain favourable responses from the general public [76]; whereby only 7% (*n* = 4) of participants consented to the pharmacist contacting the prescribing physician to discuss alternative therapies, leading to challenges in performing the intervention to its full extent [76].

#### Surveillance

An important component of AMS programmes is comprehensive surveillance of antimicrobial prescribing and resistance which provides data to focus stewardship efforts. Three studies reported on different surveillance tools, which were: a modified General Practice version of the National Antimicrobial Prescribing Survey (GP NAPS) to assess the clinical appropriateness of antimicrobial use according to local Australian guidelines [45]; a mathematical modelling of molecular testing for gonorrhoea resistance surveillance [77]; and the IQVIA Xponent antibiotic database to identify high prescribing physicians [78]. By nature, these studies did not actively involve participants, but rather served to collate relevant data through retrospective data analysis or simulation. All three tools were validated and deemed fit for integration into clinical practice to assist AMS efforts in the future [45, 77, 78].

#### National policies

Achieving AMS on a nationwide scale necessitates upstream policies and regulations, often in regard to financial aspects of prescribing. Two studies by Hammond et al. and Yin et al. provided an overview of the effects of national health policies in the United Kingdom and China, respectively [63, 68]. In 2014/15, reduced prescribing of certain antibiotics in PHC was incentivised in the UK [63], while China implemented a zero mark-up policy and a national strategy for antibiotic use [68]. In the UK, although overall antibiotic dispensation decreased 11%, the national AMS policy produced mixed results with reduced resistance of community-acquired urinary *E. coli* to amoxicillin, ciprofloxacin and trimethoprim, but increased resistance to cefalexin and co-amoxiclav [63]. China’s strategy lowered the overall antibiotic consumption by 22% and 8% for parenteral and oral antibiotics, respectively [68]. The two countries’ administrative decisions affected both urban and rural PHC centres, with differences in results between these settings delineated in the Chinese study where antibiotic consumption rates in rural PHCs fell from its peak after in 2014 whereas urban PHCs saw a steady increase in antibiotic use even after policy implementation [63, 68].

## Discussion

There is compelling evidence suggesting that there are direct correlations between excessive antibiotic prescribing and use in the PHC setting with increases in AMR rates in communities across the world. The rurality of practice plays a role in mediating antimicrobial, especially antibiotic, prescribing. Clinicians in rural and remote areas prescribed antimicrobials more frequently and rates of inappropriate prescribing are greater compared to metropolitan counterparts. Factors influencing prescribing behaviours are related to physician knowledge, attitude and behaviour, which are strongly influenced by both intrinsic and extrinsic elements. Patient factors play an important role in the development of AMR, including compliance with prescribed treatments, resources, access to healthcare, knowledge, and health literacy.

AMS strategies in the rural and remote PHC settings tend to focus on health care provider and patient education, surveillance, clinician support systems, and nationwide policy changes. These interventions resulted in largely positive outcomes with decreased rates of AMR and improved appropriateness of antimicrobial prescribing. Nonetheless, national stewardship policies also had significant impact, and based on the implementation of other health policies, have greatest impact when there are flow-on state and institutional polices that support practice.

Rural and remote settings present a unique context for PHC delivery. Many aspects can, individually or collectively, lead to inappropriate antimicrobial prescribing in these settings, from upstream factors such as inadequate workforce capacity and financial pressure to circumstances involving health care providers and patients. These factors are summarised in Table 5, together with recommendations on improving AMS implementation.

**Table 5:** Antimicrobial prescribing issues in rural and remote primary health care and actionable solutions

Factors influencing antimicrobial prescribing	Issues relevant to rural and remote settings	Potential strategies to address these issues
*Upstream factors*		
Workforce	Shortage of physicians causes excess clinical workload, which may drive increased physician fatigue. This, together with time constraints for clinical assessment from busy schedules, could impede judicious prescribing [39, 41, 46, 48]	Incentivise medical workforce to work in rural and remote regions through increasing training and specialisation opportunities as well as offering financial benefits such as higher wages and tax benefits
Remoteness and access to healthcare	Remote communities and villages are geographically isolated. A substantial proportion of residents in these regions tend to be socioeconomically disadvantaged and have difficulty in traveling long distances to larger health care centres, especially for specialist care. These physical and economical barriers result in poor health care services delivered to underserved rural and remote populations [23, 57, 58]	Appointment of a clinical champion or team aiming to provide AMS specific service to a specified geographical region. With network connectivity and infrastructure expanding to rural regions in many countries, telemedicine is a viable option for those hindered by distance to access health care. If this is not possible, remote clinics or medical camps providing subsidised medical care and prescription drugs could be set up in villages and sparsely populated areas Regular and scheduled visits from specialists could be arranged from the closest medical centres
Lack of AMR surveillance	Rural and remote populations are often underrepresented in standard surveillance systems, especially regarding antibiotic susceptibility of pathogens causing common infections [23]	Efforts to upgrade necessary technical infrastructure and establish a record-keeping culture in PHC would improve disease monitoring [23, 42]. Incentivise pathology services to compile annual antibiograms in geographic regions and to have a regional AMS team responsible for providing monitoring/surveillance activities as part of a systems approach to achieve particular targets
Financial incentives and pressures	In rural and remote health care settings, financial considerations of patient retention play a particularly important part during clinical interactions. Compounded by institutional pressures to generate revenue for health facilities and financial incentives from the sale of certain drugs, physicians may be compelled to prescribe unnecessary antimicrobials to patients who demand them [38, 52, 54]	Providing incentives for lower rates of prescribing has been proven to reduce antibiotic use Equitable access to affordable medicine, potentially through subsidised medicines or insurance
Sources of guidance	Absence or limited depth of rural-specific clinical guidelines on which PHC providers can base prescribing practices [80]	Develop clear and concise clinical guidelines in consultation with relevant stakeholders including those with AMS expertise that take into account the unique cultural and socioeconomical aspects of specific remote communities of the region. If the problem lies in their implementation, CDSS may be beneficial in translating written guidelines to clinical practice
*Health care facilities*		
Diagnostics	Rural and remote health centres lack diagnostic equipment such as X-ray, ECG and biochemistry facilities, which are often important to distinguish viral and bacterial infections and justify the correct antimicrobial prescription [38, 40]	Increase government funding and invite external sponsorship for rural and remote health care health infrastructure and equipment. Future options may include point-of-care testing for sample cultures, and antimicrobial susceptibility technologies
*Physicians*		
Deficits in diagnostic knowledge	Rural physicians often prescribe antibiotics in the face of diagnostic uncertainty, which act as a surrogate for inadequate diagnostic knowledge [38]	Studies have shown that greater adherence to guidelines for the diagnostic process translates to reduced antibiotic prescriptions [38, 51]. CDSS could provide additional guidance to physicians in addressing diagnostic and treatment uncertainty
Inadequate provider knowledge on AMR	Evidence has shown that rural and remote PHC providers with lower qualifications possess misconceptions and insufficient knowledge on the proper use of antibiotics and local resistance patterns [40, 52, 53]	Increase physician knowledge through training programmes and education campaigns [52]. CDSS may also help in this regard. If widely implemented and accepted, indirect supervision of antimicrobial prescribing by qualified professionals such as pharmacists may provide a second line of defence against inappropriate prescriptions [76] Feedback to prescribers on local resistance patterns and inappropriate prescribing may assist reflection on practice
Willingness to adjust practice for AMS	Modern measures against excessive antibiotic use such as delayed prescription is less commonly adopted by general practitioners in rural and remote areas than those in urban practices [49]	Implement policies mandating the adherence of antimicrobial prescribing practices to updated evidence-based medicine. Encourage rural physicians to attend seminars and conferences to gain exposure of the latest developments in AMS Incentivise KPI targets for appropriate prescribing for remote physicians
Concerns for patient safety	Although patients show symptoms indicative of a viral infection during the initial consultation, rural physicians tend to prescribe antibiotics due to the fear of complications arising from secondary bacterial infections, which may occur at a time when the patient is unable to access medical care [52, 54]	For clinical cases involving ambiguity in aetiology, encourage physicians to employ a watch-and-wait strategy with appropriate follow up and management [73]. Schedule regular home visits for patients at risk of being lost to follow-up
Pressure to maintain good patient relations	Physicians often need to maintain a good reputation among members of the closely-knit rural community and are highly dependent on patient relations to maintain personal livelihoods. Due to their relatively low position in health care networks, rural physicians are especially vulnerable to medico-legal disputes, hence they would often fulfil their patients’ wishes regarding antimicrobial prescriptions [54]	Establish formal councils and committees to advocate and protect the interests of rural doctors. Perform clinician education on the importance of appropriate prescribing and engaging with patients in discussions on judicious use of antimicrobial agents, especially antibiotics
*Pharmacists and informal health care providers*		
Over-the-counter antimicrobial agents	Particularly in developing countries, unlicensed practitioners and pharmacists are often the primary source of health care. In rural and remote areas with a shortage of primary health physicians, these providers provide consultations and supply antibiotics to rural communities, often without a prescription [40, 55, 58]	Enforce stricter rules and regulations on the provision of antibiotics to the general public, restricting their availability to those with a prescription by a qualified health professional. Provide education to health care professionals on the dangers of AMR and implications of excessive use of antimicrobials
*Patients*		
Carriage of potentially pathogenic microorganisms	Nasopharyngeal carriage of respiratory pathogens is found to be significantly higher in rural and remote populations than those in urban areas, which portends a higher risk of transmitting organisms resistant to antibiotics [30, 30]	Monitor populations at risk of bacterial infestation Consider decolonisation therapy using topical agents in high risk patients Perform community-wide surveillance on carriage of drug-resistant organisms in areas where AMR is suspected to be problematic
Suboptimal adherence	Rural and remote populations, especially those in lower socio-economic status groups, generally have a poorer adherence to prescribed treatment [58]	Physicians and allied health professionals need to adopt a patient-centred approach that addresses factors leading to non-adherence and provide clear instructions for patients to follow regarding prescribed medications
Self-medication	Given the distance to PHC centres and costly consultation fees, patients tend to develop the habit of self-medication which includes using over-the-counter medications or those leftover from past illnesses or obtained from friends and family members [59]	Implement community-based programmes and campaigns to promote help-seeking behaviour and educate the public on the hazards and risks of self-medication Restrict inappropriate access to antibiotics
Expectation of an antimicrobial prescription	Some population groups have higher expectations of obtaining antibiotics after each consultation and may prefer to visit doctors who prescribe antibiotics [47, 52]	Provide education to patients during consultations through easy-to-understand explanations and distribution of printed materials on proper antibiotic use—preferably using the patient’s own language and tailored to local cultures and preferences [57] Implement community-based programmes and campaigns to promote proper antibiotic use
Patient knowledge	Populations in rural and remote areas are relatively disadvantaged in accessing health information. Evidence have shown that antimicrobial prescriptions are more likely to be given to patients who have lower antimicrobial-related knowledge [47, 59]	Conduct community-level stewardship programmes focused on community health literacy [60, 61]

*AMR* antimicrobial resistance, *AMS* antimicrobial stewardship, *CDSS* clinical decision support system, *PHC* primary health care

Previous research has attempted to provide guidance in relation to effective AMS activities to address the specific needs of rural and remote primary health care. Bowen et al. [11] highlighted the challenges of AMS programmes in the context of the Australian Indigenous population and the need to coordinate with relevant sectors such as housing, environmental health and education to improve health-related literacy and infrastructure in order to contain the spread of resistant pathogens. Such a programme should strive to review and improve antimicrobial prescribing and use, apply AMR surveillance data, and inform clinicians and regulatory bodies on judicious antimicrobial use.

In a systematic review of 39 studies in primary care, Arnold et al. [79] found that multifaceted interventions involving educating physicians, patients and the public via a range of different formats across various health care and community settings were most successful in lowering rates of inappropriate antimicrobial prescribing. Although the systematic review included all studies in ambulatory care and not strictly rural or remote settings, it concluded that a single intervention for all contexts does not exist [79]. There is no one-size-fits-all approach to AMS and targeted interventions for specific groups and varied contexts are necessary for effective AMS programmes to prevent AMR emergence.

The review identified several gaps in the literature around AMS in remote and rural PHC. First, the existing research and evidence as to the effectiveness of AMS interventions is quite short-term. Longer term studies, of years rather than week to months, are necessary to increase reliability of findings, given that AMR evolves over a period of time through exchange of genetic material driven by selection pressure [1, 22]. Despite the absence of an all-encompassing AMS intervention, efforts to delineate the most effective components of each intervention would contribute towards developing the best strategy for a given target population. The patient voice, particularly rural and remote populations, is largely absent from the discussion. A greater focus on understanding the patient perspective and investigation of patient understanding of AMR may provide greater success in implementing community-based education programmes. Finally, we acknowledge a distinction exists between rural and remote health [17], but this was not reflected in current literature. Our review involved only five studies on the remote Indigenous community; four in Australia [29, 30, 45, 77] and one in Canada [23]. How AMS strategies differ between rural and remote areas, as well as their applications and significance towards Indigenous populations, warrant further investigation.

### Limitations and strengths of this review

Despite efforts to thoroughly search for and examine articles, this is not a systematic review and a quantitative statistical analysis could not be conducted due to the variability in measured outcomes and target populations. There is a possibility of language bias as the search was limited to the English language. Additionally, all research studies in this field were not covered, especially those conducted prior to the year 2000. Lastly, some of the papers did not separate urban settings from rural and remote settings, so distinct lessons for the rural and remote settings are harder to identify.

Nonetheless, to the best of our knowledge, this is the first structured review to examine AMS in rural and remote PHC. It included a respectable number of studies from around the world. Screening and appraisal of articles were performed by at least two independent researchers, thereby reducing inter-rater variability. Relevant data extracted from studies were categorically summarised in tables for rapid search, thus enabling a comprehensive description of recent evidence on antimicrobial resistance and stewardship in rural and remote PHC settings.

## Conclusion

There is strong evidence that excessive antimicrobial prescribing and use greatly contribute to the rise of AMR. Various physician and patient factors are at play, especially in PHC in remote and rural settings where health resources and regulations are limited. Several AMS strategies aimed at education, clinical support, surveillance, and policies have been attempted with mostly positive results. Future researchers should place emphasis on investigating the effectiveness of interventions over longer durations, capturing the patient perspective and contrasting AMS in rural and remote settings.

## Supplementary Information

**Additional file 1.** Critical appraisal of included articles based on Joanna Briggs Institute (JBI) checklists.

## Data Availability

All data generated or analysed during this study are included in this published article (and its Additional file 1).

## Abbreviations

AD: Academic detailing
AMR: Antimicrobial resistance
AMS: Antimicrobial stewardship
CDSS: Clinical-decision support system
CI: Confidence interval
GP NAPS: General Practice version of the National Antimicrobial Prescribing Survey
MMM: Modified mondahs model
MRSA: Methicillin-resistant *Staphylococcus aureus*
OR: Odds ratio
PBL: Problem-based learning
PHC: Primary health care
RCT: Randomised controlled trial
