# Supplementary material for: Antimicrobial stewardship in rural and remote primary health care: a narrative review
Source: Antimicrob Resist Infect Control. 2021 Jul 13;10:105. doi: 10.1186/s13756-021-00964-1 (PMC8278763; doi:10.1186/s13756-021-00964-1)
Supplement: Supplementary file 1 — Additional file 1. Critical appraisal of included articles based on Joanna Briggs Institute (JBI) checklists. [file 13756_2021_964_MOESM1_ESM.docx]

**Table S1.** Critical Appraisal (Cross Sectional Study)

| **Study details, country** | **Were the criteria for inclusion in the sample clearly defined?** | **Were the study subjects and the setting described in detail?** | **Was the exposure measured in a valid and reliable way?** | **Were objective, standard criteria used for measurement of the condition?** | **Were confounding factors identified?** | **Were strategies to deal with confounding factors stated?** | **Were the outcomes measured in a valid and reliable way?** | **Was appropriate statistical analysis used?** | **Results** |
| --- | --- | --- | --- | --- | --- | --- | --- | --- | --- |
| Barker et al. (2017), India | Yes | Yes | Yes | Yes | Yes | Unclear | Yes | Unclear | 6 Yes 2 Unclear |
| Boada et al. (2018), Spain | Yes | Yes | Yes | Unclear | Yes | Yes | Yes | Yes | 7 Yes 1 Unclear |
| Chai et al. (2019), China | Yes | Yes | Yes | Yes | Yes | Yes | Yes | Yes | 8 Yes |
| Chen et al. (2020), China | No | Yes | Unclear | Unclear | Yes | Yes | Yes | Yes | 5 Yes 1 No 2 Unclear |
| Davey et al. (2020), Australia | No | Yes | Unclear | Yes | Yes | Yes | Yes | Yes | 7 Yes 1 Unclear |
| Homaidan and Issam (2018), Saudi Arabia | No | Yes | Yes | Yes | Yes | Unclear | Yes | Yes | 6 Yes 1 No 1 Unclear |
| Kumar et al. (2008), India | Yes | Yes | Yes | Yes | Yes | Yes | Yes | Yes | 8 Yes |
| Kumari Indira et al. (2008), India | Yes | Yes | Yes | Yes | Unclear (might be yes) | Yes | Yes | Yes | 7 Yes 1 Unclear |
| Kwiatkowska et al. (2020), China | Yes | Yes | Yes | Yes | Unclear | Yes | Yes | Yes | 7 Yes 1 Unclear |
| Nair et al. (2019), India | Yes | Yes | N/A | N/A | Unclear | Unclear | Yes | Yes | 4 Yes 2 N/A 2 Unclear |
| Rhee et al. (2019), Kenya | Yes | Yes | Unclear | Yes | Yes | Yes | Yes | Yes | 7 Yes 1 Unclear |
| Salm et al. (2018), Germany | Yes | Yes | Yes | Yes | Yes | Yes | Yes | Yes | 8 Yes |
| Sarwar et al. (2018), Pakistan | Yes | Yes | Unclear | Yes | Yes | Yes | Yes | Yes | 7 Yes 1 Unclear |
| Wang et al. (2014), China | No | Yes | Yes | Yes | Unclear | No | Yes | Yes | 6 Yes 1 No1 Unclear |
| Wang et al. (2020), China | Yes | Yes | Yes | Yes | Yes | Yes | Yes | Yes | 8 Yes |
| Wood et al. (2007), Wales | No | Yes | Unclear | Yes | Yes | Yes | Yes | Yes | 6 Yes 1 No1 Unclear |
| Yuguero et al. (2019), Spain | No | Yes | Yes | Yes | Yes | Yes | Yes | Yes | 7 Yes 1 No |
| Zhang et al. (2016), China | Yes | Yes | Unclear | Yes | Yes | Yes | Yes | Yes | 7 Yes 1 Unclear |

**Table S2.** Critical Appraisal (Systematic Review)

| **Study details, location** | **Is the review question clearly and explicitly stated?** | **Were the inclusion criteria appropriate for the review question?** | **Was the search strategy appropriate?** | **Were the sources and resources used to search for studies adequate?** | **Were the criteria for appraising studies appropriate?** | **Was critical appraisal conducted by two or more reviewers independently?** | **Were there methods to minimize errors in data extraction?** | **Were the methods used to combine studies appropriate?** | **Was the likelihood of publication bias assessed?** | **Were recommendations for policy and/or practice supported by the reported data?** | **Were the specific directives for new research appropriate?** | **Results** |
| --- | --- | --- | --- | --- | --- | --- | --- | --- | --- | --- | --- | --- |
| Costelloe et al. (2010), multiple | Yes | Yes | Yes | Yes | Yes | Yes | Yes | Yes | Yes | Yes | Yes | 11 Yes |
| Evans et al. (2019), multiple | Yes | Yes | Yes | Yes | Unclear | Unclear | Yes | Yes | No | Yes | Unclear | 7 Yes 1 No 3 Unclear |
| Hansen et al. (2019), multiple | Yes | Yes | Yes | Yes | Unclear | Yes | Yes | Yes | Yes | Yes | Yes | 10 Yes 1 Unclear |

**Table S3.** Critical Appraisal (Quasi-Experimental Studies)

| **Study designs, Location** | **Is it clear in the study what is the ‘cause’ and what is the ‘effect’?** | **Were the participants included in any comparisons similar?** | **Were the participants included in any comparisons receiving similar treatment/care, other than the exposure or intervention of interest?** | **Was there a control group?** | **Were there multiple measurements of the outcome both pre and post the intervention/exposure?** | **Was follow up complete and if not, were differences between groups in terms of their follow up adequately described and analyzed?** | **Were the outcomes of participants included in any comparisons measured in the same way?** | **Were outcomes measured in a reliable way?** | **Was appropriate statistical analysis used?** | **Results** |
| --- | --- | --- | --- | --- | --- | --- | --- | --- | --- | --- |
| Belongia et al. (2001), United States | Yes | Unclear | Yes | Yes | Yes | Yes | Yes | Yes | Yes | 8 Yes 1 Uncelar |
| Chiswell et al. (2019), United States | Yes | Yes | Yes | No | Yes | No | Yes | Yes | Yes | 7 Yes 2 No |
| Cummings et al. (2020), United States | Yes | Unclear | Yes | No | Yes | Yes | Yes | Yes | Unclear | 6 Yes 2 Unclear 1 No |
| Haenssgen et al. (2018), Laos | Yes | Unclear | Yes | Yes | Yes | Unclear | Yes | Yes | Yes | 7 Yes 2 Unclear |
| Mandaras-Kelly et al. (2006), United States | Yes | Yes | Yes | Yes | Yes | Yes | Yes | Yes | Unclear | 8 Yes 1 Uncelar |
| Xue et al. (2019), China | Yes | Yes | Yes | Yes | Yes | Yes | Yes | Yes | Yes | 9 Yes |

**Table S4.** Critical Appraisal (Qualitative Research)

| **Study designs, Location** | **Is there congruity between the stated philosophical perspective and the research methodology?** | **Is there congruity between the research methodology and the research question or objectives?** | **Is there congruity between the research methodology and the methods used to collect data?** | **Is there congruity between the research methodology and the representation and analysis of data?** | **Is there congruity between the research methodology and the interpretation of results?** | **Is there a statement locating the researcher culturally or theoretically?** | **Is the influence of the researcher on the research, and vice- versa, addressed?** | **Are participants, and their voices, adequately represented?** | **Is the research ethical according to current criteria or, for recent studies, and is there evidence of ethical approval by an appropriate body?** | **Do the conclusions drawn in the research report flow from the analysis, or interpretation, of the data?** | **Result** |
| --- | --- | --- | --- | --- | --- | --- | --- | --- | --- | --- | --- |
| Collins et al. (2020), United States | Yes | Yes | Yes | Yes | Yes | No | No | N/A | N/A | Yes | 6 Yes 2 No 2 N/A |
| Dallas et al. (2014), Australia | Yes | Yes | Yes | Yes | Yes | No | No | Yes | Yes | Yes | 8 Yes 2 No |
| Duane et al. (2016), Ireland | Yes | Yes | Yes | Yes | Yes | No | No | Yes | Yes | Yes | 8 Yes 2 No |
| Haenssgen et al. (2018), Laos | Yes | Yes | Yes | Yes | Yes | No | No | Yes | Yes | Yes | 8 Yes 2 No |
| Salm et al. (2018), Germany | Yes | Yes | Yes | Yes | Yes | No | No | Yes | Yes | Yes | 8 Yes 2 No |

**Table S5.** Critical Appraisal (Prevalence Study)

| **Study designs, location** | **Was the sample frame appropriate to address the target population?** | **Were study participants sampled in an appropriate way?** | **Was the sample size adequate?** | **Were the study subjects and the setting described in detail?** | **Was the data analysis conducted with sufficient coverage of the identified sample?** | **Were valid methods used for the identification of the condition?** | **Was the condition measured in a standard, reliable way for all participants?** | **Was there appropriate statistical analysis?** | **Was the response rate adequate, and if not, was the low response rate managed appropriately?** | **Results** |
| --- | --- | --- | --- | --- | --- | --- | --- | --- | --- | --- |
| Albrich et al. (2004), United States and European countries | Unclear | Unclear | Yes | No | N/A | Yes | Yes | Yes | Yes | 5 Yes 1 No 2 Unclear 1 N/A |
| Cuningham et al. (2020), Australia | Yes | Yes | Yes | Yes | Yes | Yes | Yes | Yes | Yes | 9 Yes |
| Giles et al. (2019), United States | Yes | Yes | No | Yes | N/A | Yes | Yes | Unclear | N/A | 5 Yes 1 No 1 Unclear 2 N/A |
| Hammond et al. (2020), United Kingdom | Yes | Yes | Yes | No | N/A | Yes | Yes | Yes | N/A | 6 Yes 1 No 2 N/A |
| Jeong et al. (2020), Canada | Yes | Yes | Yes | Yes | Yes | Yes | Yes | Yes | Yes | 9 Yes |
| Silverman et al. (2017), Canada | Yes | Yes | Yes | Yes | Yes | Yes | Yes | Yes | Yes | 9 Yes |
| Staub et al. (2020), United States | Yes | Yes | Yes | Yes | N/A | Yes | Yes | Yes | N/A | 7 Yes 2 N/A |
| Van Bijnen et al. (2015), European countries | Yes | Yes | Yes | Yes | N/A | Yes | Yes | Yes | Yes | 8 Yes 1 N/A |
| Yin et al. (2018), China | Yes | Yes | Yes | Yes | N/A | N/A | Yes | Yes | N/A | 6 Yes 3 N/A |

**Table S6.** Critical Appraisal (Cohort Study)

| **Study designs, Location** | **Were the two groups similar and recruited from the same population?** | **Were the exposures measured similarly to assign people to both exposed and unexposed groups?** | **Was the exposure measured in a valid and reliable way?** | **Were confounding factors identified?** | **Were strategies to deal with confounding factors stated?** | **Were the groups/participants free of the outcome at the start of the study (or at the moment of exposure)?** | **Were the outcomes measured in a valid and reliable way?** | **Was the follow up time reported and sufficient to be long enough for outcomes to occur?** | **Was follow up complete, and if not, were the reasons to loss to follow up described and explored?** | **Were strategies to address incomplete follow up utilized?** | **Was appropriate statistical analysis used?** | **Results** |
| --- | --- | --- | --- | --- | --- | --- | --- | --- | --- | --- | --- | --- |
| Hare et al. (2013), Australia & Alaska | Yes | Yes | Yes | Yes | Yes | Yes | Yes | Yes | Yes | N/A | Yes | 10 Yes 1 N/A |
| Singer et al. (2018), Canada | Yes | Yes | Yes | Yes | Yes | Yes | Yes | N/A | N/A | N/A | Yes | 8 Yes 3 N/A |

**Table S7.** Critical Appraisal (Randomized Controlled Trial)

| **Study design, location** | **Was true randomization used for assignment of participants to treatment groups?** | **Was allocation to treatment groups concealed?** | **Were treatment groups similar at the baseline?** | **Were participants blind to treatment assignment?** | **Were those delivering treatment blind to treatment assignment?** | **Were outcomes assessors blind to treatment assignment?** | **Were treatment groups treated identically other than the intervention of interest?** | **Was follow up complete and if not, were differences between groups in terms of their follow up adequately described and analyzed?** | **Were participants analyzed in the groups to which they were randomized?** | **Were outcomes measured in the same way for treatment groups?** | **Were outcomes measured in a reliable way?** | **Was appropriate statistical analysis used?** | **Was the trial design appropriate, and any deviations from the standard RCT design (individual randomization, parallel groups) accounted for in the conduct and analysis of the trial?** | **Results** |
| --- | --- | --- | --- | --- | --- | --- | --- | --- | --- | --- | --- | --- | --- | --- |
| Doan et al. (2020), Niger | Yes | Yes | Yes | Yes | Unclear | Unclear | Yes | Yes | Yes | Yes | Yes | Yes | Yes | 11 Yes 2 Unclear |
| Gonzales et al. (2013), United States | Unclear | Yes | Unclear | Unclear | No | Yes | Yes | Yes | Yes | Yes | Yes | Yes | Yes | 9 Yes 1 No 3 Unclear |
| Hare et al. (2015), Australia & New Zealand | Unclear | Yes | Yes | Yes | No | Yes | Yes | Yes | Yes | Yes | Yes | Yes | Yes | 11 Yes 1 No 1 Unclear |
| Hoberman et al. (2016), United States | Unclear | Yes | Yes | Yes | Yes | Unclear | Yes | Yes | Yes | Yes | Yes | Yes | Yes | 11 Yes 2 Unclear |
| Little et al. (2001), England | Yes | No | Yes | No | No | No | Yes | Yes | Yes | Yes | Yes | Yes | Yes | 9 Yes 4 No |
| Rubin et al. (2006), United States | Unclear | Unclear | Unclear | Unclear | Unclear | Unclear | Yes | Yes | Yes | Yes | Yes | Yes | Yes | 7 Yes 6 Unclear |
| Samore et al. (2005), United States | Unclear | Unclear | Unclear | Unclear | Unclear | Unclear | Yes | Yes | Yes | Yes | Yes | Yes | Yes | 7 Yes 6 Unclear |
| Varonen et al. (2007), Finland | Yes | No | Unclear | No | No | Unclear | Yes | Yes | Yes | Yes | Yes | Yes | Yes | 8 Yes 3 No 2 Unclear |
| Wei et al. (2017), China | Yes | Yes | Yes | Yes | No | Unclear | Yes | Yes | Yes | Yes | Yes | Yes | Yes | 11 Yes 1 No 1 Unclear |
| Wei et al. (2019), China | Yes | Yes | Yes | Yes | No | Unclear | Yes | Yes | Yes | Yes | Yes | Yes | Yes | 11 Yes 1 No 1 Unclear |
| Zhang et al. (2018), China | Yes | No | Unclear | No | No | Unclear | Yes | Yes | Yes | Yes | Yes | Yes | Yes | 8 Yes 3No 2 Unclear |

**Table S8.** Critical Appraisal (Diagnostic Test Accuracy Study)

| **Study designs, Location** | **Was a consecutive or random sample of patients enrolled?** | **Was a case control design avoided?** | **Did the study avoid inappropriate exclusions?** | **Were the index test results interpreted without knowledge of the results of the reference standard?** | **If a threshold was used, was it pre-specified?** | **Is the reference standard likely to correctly classify the target condition?** | **Were the reference standard results interpreted without knowledge of the results of the index test?** | **Was there an appropriate interval between index test and reference standard?** | **Did all patients receive the same reference standard?** | **Were all patients included in the analysis?** | **Results** |
| --- | --- | --- | --- | --- | --- | --- | --- | --- | --- | --- | --- |
| Hui et al. (2015), Australia | Unclear | Yes | No | Yes | Unclear | Yes | Yes | Yes | Yes | Yes | 7 Yes 1 No 2 Unclear |
| Schwartz et al. (2019), Canada | Yes | N/A | Yes | Unclear | N/A | Yes | Unclear | Yes | Yes | Yes | 6 Yes 2 N/A 2 Unclear |
